# Biodegradable functionalized magnetite nanoparticles as binary-targeting carrier for breast carcinoma

**DOI:** 10.1186/s13065-023-00915-4

**Published:** 2023-02-13

**Authors:** Magda Ali Akl, Amira Mostafa Kamel, Mahmoud Ahmed Abd El-Ghaffar

**Affiliations:** 1 Chemistry Department, Faculty of Science, Mansoura University, Mansoura, Egypt; 2 Polymers and Pigments Department, National Research Centre, 33-El-Bohouth St. Dokki, Cairo, Egypt

**Keywords:** Superparamagnetic magnetite nanoparticles, Doxorubicin hydrochloride, Binary targeting drug system, Breast carcinoma

## Abstract

In this study, Superparamagnetic magnetite nanoparticles (SPMNPs) are used in a new way as direct nanocarrier for Doxorubicin hydrochloride (DOX) via the functionalization of their surface with tri-sodium citrate through ligand exchange to conjugate DOX with imine bond to form tri-sodium citrate functionalized magnetite loaded DOX nanoparticles (DOX/Cit-MNPs). The DOX/Cit-MNPs were coated with chitosan to form chitosan coated citrate functionalized magnetite loaded DOX nanoparticles (Cs/DOX/Cit-MNPs) to offer biodegradability and pH-sensitive drug release features. The Fourier transform infrared spectroscopy (FTIR) analysis confirmed functionalization of SPMNPs, DOX-conjugation, and chitosan coating. The trans electron microscopy (TEM) show spherical nanostructures with average size 40 nm for coated nanocarriers. The saturation magnetization value of carrier was 59 emu/g.The in-vitro release of DOX from the chitosan coated tri-sodium citrate functionalized magnetite loaded DOX nanoparticles (Cs/DOX/Cit-MNPs) was studied to be 75% at pH 5.5 and 28.6% at pH 7.4 which proves the pH sensitivity of encapsulated Cs/DOX/Cit-MNPs. The effect of Cs/DOX/Cit-MNPs toward Human Breast Cancer Cell lines (MCF7) was studied and found to be 76% without magnet and 98% with external magnet after 72 h. With increasing DOX concentration and treatment time, the cell inhibition (IR%) of DOX solution and Cs/DOX-Cit-MNPs suspension to all cells is increased. Cs/DOX/Cit-MNPs showed sustained release and good inhibition to cancer cells and offer a protective mode for normal cells (WISH) compared to the free DOX.

**Graphical Abstract:**

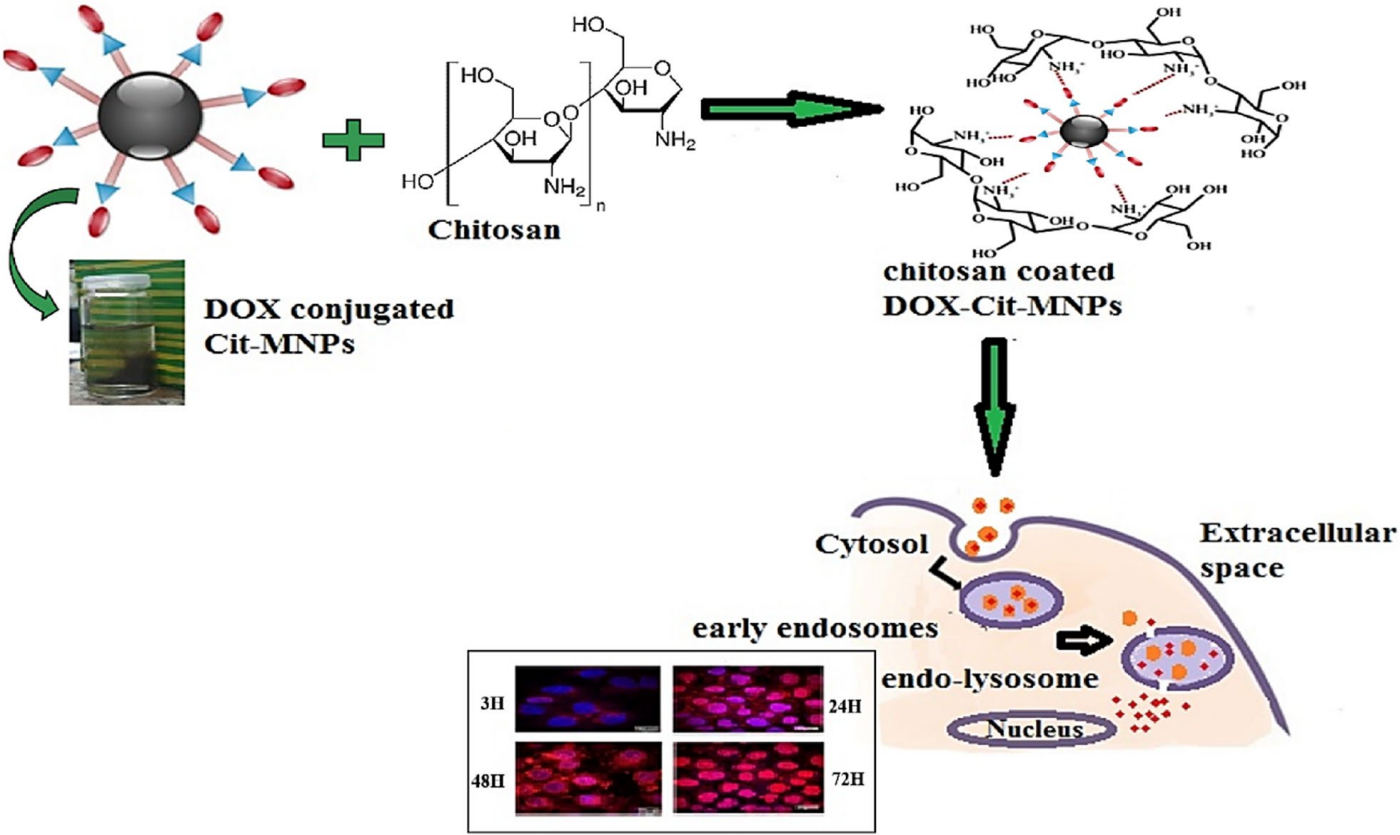

**Supplementary Information:**

The online version contains supplementary material available at 10.1186/s13065-023-00915-4.

## Introduction

Recently, the expansion of nano-medical technology has been observed mainly in the fields of nano pharmaceutics, drug delivery and targeted therapy which represent one of the most promising options for many diseases especially for cancer.

Cancer is the second deadly disease in the world that expects to surpass heart disease as the top cause of death in the coming few years [1]. In 2015, number of cases diagnosed with cancer was 1658.37 in the United States [1] and 196,900 in Canada and a quarter of these cases are expected to die [2]. Besides the unknown causes of cancer, its treatment is exceptionally challenging. The available treatments are single or combination of chemotherapies, radiation, and surgery; all of which are not ensured to be actually effective. Chemotherapy and radiation intend to destroy cancer cells; yet they have significant side effects on healthy cells and if the infected cells are targeted, the anticancer drug release rate is usually uncontrolled [3].

Doxorubicin (DOX), as an anti-cancer drug model, is one of the anthracycline family and antineoplastic agent which is considered effective for many carcinoma like leukemia, liver cancer and is very effective in advanced stages of breast cancer [4]. DOX has many side effects, especially heart damaging and also low stability in the circulatory system [5].

A lot of the unfavorable side effects of anti-cancer drugs specially DOX can be overcome by targeting the drugs and directing them to tumor sites which increase their cytotoxic effect against cancer cells [6].

Many targeting techniques have been evaluated; one of these techniques is Nano-enabled drug delivery technique (NEDD) which is concerned with specific cell targeting to promote drug release in the infected site [7]. The NEDD systems include but not limited to nanoparticles, nanofibers, polymers, nanocapsules, quantum dots and carbon nanotubes [8].

One of the NEDD systems that are offering a promising concept for treating tumors is Magnetic drug targeting (MDT). Magnetic nanoparticles are not only a well-known category of NEDD systems, but also they are FDA approved and have been applied in many medical fields as drug delivery, gene therapy, magnetic resonance imaging (MRI), tissue repair, and biosensors [9]. A distinct type of magnetite is the superparamagnetic iron oxide nanoparticles (SPMNPs) which are most commonly used for biomedical applications due to their specific features like: biocompatibility and ease of synthesis [10]. The properties of SPMNPs depend on the surface nature. So, surface chemistry plays important role in its biological and chemical properties when using in biomedical applications.

As every therapeutic regimen has its advantages and disadvantages, the magnetic drug targeting (MDT) system has some disadvantages. Hence, depending on the physical and chemical properties of SPMNPs, surface functionalization and modification are considered one of the best solutions not only to overcome the disadvantages of SPMNPs but also, to improve their features and make them suitable for a wide range of applications.

Iron oxide NPs (IONPs) especially magnetite (Fe_3_O_4_) have been widely scrutinized in the medical fields. IONPs can reach the malignant tissue/cells in a (i) passive manner, e.g., by the enhanced permeability retention effect (EPR), (ii) an active manner by applying ligands, specific-cell-targeting, and (iii) an extraneous manner where an external stimulus, e.g., US controls the cellular uptake and the release of neoplastic cargo. One of the challenges of using IONPs is that they tend to agglomerate because of their larger surface area-to-volume ratio and dipolar coupling. The alterations with biologically compatible materials can prevent agglomeration and improve their stability, biocompatibility, dispersibility, biodistribution, and blood circulation time (BCT) [11]. Recently, numerous stimuli responsive smart MNPs have been engineered to deliver therapeutic cargo in response to any stimulant including pH, temperature, redox, MF, etc. [12–16]. Their advantages include potential higher drug accumulation in targeted organs, prolonged BCT, enhanced systemic stability, decreased toxic side effects towards normal cells, and improved therapeutic efficacy [17, 18]. However, their safety, large-scale manufacturing challenges, cost-effectiveness, and poor perception of disease heterogeneity in the patient population constrain their clinical translation.

Despite a vast number of polymeric materials, chitosan NPs have broadly been studied due to their exclusive chemical properties and applications [19]. Chitosan is a linear carbohydrate-backboned biopolymer that contains *N*-acetyl-d-glucosamine and d-glucosamine repeating units. Owing to its active amino groups, chitosan could be named as a versatile biopolymer. In addition to chitosan’s antimicrobial activity, mucoadhesivity, and antitumor activity, it could be used as a great drug carrier [16]

There are two methods for surface functionalization: ligand addition and ligand exchange [20, 21]. In ligand exchange, original surface replaced with certain functional groups as thiol, carboxylic acid, amine, which improves the surface properties [22]. For ligand addition, polymers adsorb on surface of the SPION particles physically by hydrophobic interactions, electrostatic and/or by hydrogen bonding. The nature of the polymer may be synthetic e.g. polyethylene glycol, polyacrylic acid, poly (vinyl pyrrolidone), poly (vinyl alcohol), poly (methacrylic acid) or natural, as in the case of chitosan, starch, cellulose, agarose, and dextran. Natural polymers, especially polysaccharide, are widely used in the field of drug delivery and biomedical applications [23]. Chitosan, the second most abundant biopolymer after cellulose, is an inexpensive, inert, hydrophilic, biocompatible support, and is thus attractive for drug delivery systems [24, 25].

The objectives of the present study can summarized as follows:

i. Design of SPMNPs as a drug carrier for binary targeting of DOX by functionalizing the surface of SPMNPs with COOH groups from tri-sodium citrate.
ii. Characterization of the SPMNPs using different characterization techniques viz. FTIR, Zeta potential, SEM/TEM, EDX, XDR Saturation magnetization (MS) values of MNPs
iii. Elucidation of the reaction mechanism of Fe_3_O_4_ nanoparticles with tri-sodium citrate, the reaction mechanism of DOX with Cit-MNPs and the reaction mechanism of chitosan with DOX-Cit-MNPs
iv. Assessment of DOX loading efficiency and the in-vitro release studies of DOX from Cs/DOX/Cit-MNPs
v. Evaluation of the Cellular Internalization of Cs/DOX-Cit-MNPs
vi. Studying the cytotoxicity potentials of Cs/DOX/Cit-MNPs against cancerous and normal cells.

## Material and methods

### Materials

Iron (III) chloride hexahydrate (FeCl_3_.6H_2_O), Iron (II) chloride tetra hydrate (FeCl_2_.4H_2_O) were purchased from Sigma. Tri-sodium citrate from (Fluka). Chitosan (Cs) powder was a product of Sigma-Aldrich and the degree of deacetylation (DD) was 87%. Sodium tripolyphosphate (STPP) with a purity of 85%. Glacial acetic acid (≥ 99.85%), sodium hydroxide ≥ 98% beads and doxorubicin hydrochloride (DOX. HCl) were purchased from Sigma Aldrich.

### Preparations

#### Synthesis of MNPs

In three-neck flask, MNPs were synthesized by co-precipitation of Fe^2+^ and Fe^3+^ solution with ratio (2:1) at 70 °C under N_2_ [26].

At pH 10, Fe_3_O_4_ nanoparticles are precipitated by adding NaOH solution (30%) under vigorous stirring at 80 °C. With external magnet, the black precipitate was separated, washed several times with distilled water and ethanol. Finally, Fe_3_O_4_ is dried overnight under vacuum at 50 °C.

$${\text{Fe}}^{{ + {2}}} + {\text{ 2Fe}}^{{ + {3}}} + {\text{ 8Cl}}^{ - } + {\text{ 8NaOH}}\,\buildrel {\rm{pH}\;10} \over \longrightarrow\,\;{\text{Fe}}_{{3}} {\text{O}}_{{4}} + {\text{ 8NaCl }} + {\text{ 4H}}_{{2}} {\text{O}}{.}$$

#### Preparation of Cit-MNPs

In three necked flask, inert atmosphere under continuous N_2_ flow, mixture of FeCl_2_ 0.7H_2_O, dehydrated Fe_2_Cl_3_ and tri- sodium citrate (Na_3_C_6_H_5_O_7_) solutions [27] with molar ratio Fe_3_^+^:Fe_2_^+^:Na_3_C_6_H_5_O_7_ (2:1:0.5) are mixed drop wising in NaOH solution with 30% concentration at 80 °C under vigorous stirring and pH10 for 3 h. With external magnet, the black precipitate of MNPs modified with Na_3_C_6_H_5_O_7_ tri-sodium citrate (Cit-MNPs) was separated and washed several times with distilled water followed by ethanol. Finally, the Cit-MNPs are dried overnight under vacuum at 50 °C.

#### Preparation of DOX/Cit-MNPs

30% (W:W) of DOX was added to 0.5 g of Cit-MNPs suspended in distilled water with concentration (10 mg/ml) under nitrogen and vigorous stirring at 80 °C for 3 

With external magnet the black precipitate was separated, the particles were washed several time with double-distilled water and then dried under vacuum at 50 °C for 24 h to get the black powder.

#### Preparation of Cs/DOX/Cit-MNPs

Under N_2_, a suspension from DOX loaded Cit-MNPs (50 mg) is stirred at room temperature in three necked flask with Cs solution (3 mg/ml). Then, STPP solution (1 mg/ml) is added drop wise to this mixture and left to stand for 3 h.

The dark suspension is separated by magnet, washed several times by double-distilled water and dried in vacuum oven at 50^◦^C overnight.

### Determination of loading efficiency

The loading efficiency (LE%) was calculated indirectly by detecting the absorbance of unloaded drug in the supernatant with a UV-spectrophotometer for DOX, at 481 nm (Eq. 1) before and after coating MNPs & Cit-MNPs with chitosan layer.

1 $$\mathrm{Drug\,L}.\mathrm{E\, \% }= \frac{\mathrm{Total\,drug}-\mathrm{Free\,drug}}{\mathrm{Total\,drug}} \times 100.$$

### In vitro drug release studies

In vitro release profiles of DOX from Cs/DOX-Cit-MNPs were determined as follows: 3 ml of Cs/DOX-Cit-MNPs suspension was withdrawn and dialyzed in a dialysis membrane tube (MW 12,000 to 14,000 Da) against 50 ml of 0.1 M buffer in two different pHs (pH 5.0: acetate and 7.4: phosphate) under shaking at 100 rpm at 37 °C. Repeat this process after 6, 12, 24, 48, 72,…168 h and replace the withdrawn sample by 3 mL fresh medium. The amount of released drug was determined by UV–vis spectrometry at 481 nm in comparison to the standard curve.

### Characterization

#### Fourier transform infrared (FTIR) spectra studies

FTIR spectra of the MNPs, Cit-MNPs, DOX/Cit-MNPs and Cs/DOX/Cit-MNPs were recorded by FT-IR spectrophotometer Bruker Vector 22 Germany in the range 400 to 4000 cm^−1^ at resolution of 4 cm^−1^.

#### Zeta potential

The Zeta potential was determined by the Malvern Zetasizer nano s in deionized water at (pH 6.3, ionic strength 0) and 10^−3^ M NaCl aqueous solutions at pH 4–9 (adjusted by NaOH or HCl) with concentration (1 mg/ml).

#### Transmission electron microscopy (TEM)

To determinate particle size and shape of Cit-MNPs, DOX/Cit-MNPs and Cs/DOx-Cit-MNPs, TEM was used and they were negatively stained with 1.0% (w/v) phosphotungstic acid.

#### Scanning electron microscopy (SEM)

The morphological characteristics of synthesized nanoparticles were examined by SEM. The samples were coated with gold and imaging is performed at accelerating voltage 30 kV.

#### Energy dispersive X-ray spectroscopy (EDX)

EDX analysis was carried out to confirm the functionalization of MNPs surface by determination the percentage of COOH and NH_2_ groups on the functionalized magnetite nanoparticles.

#### Thermal gravimetric analysis (TGA)

To study the thermal stability of Cs-MINPs under temperature range (0–600 °C), thermogravimetric analysis for Cs/DOX-Cit-MNPs was carried out with Shimadzu TGA-50H. Analyses were carried out with 20 mg of sample under nitrogen atmosphere with heating at 5 °C/min.

#### X-ray diffraction (XRD)

The crystal structures of the nanoparticles were detected by XRD.

#### Saturation magnetization (MS) values of MNPs

The magnetization force of MNPs was determined by (VSM) device magnetometer at 25 °C and ± 10,000G applied magnetic field.

### Cellular internalization of Cs/DOX-Cit-MNPs

MCF-7 cell line was used to study the cellular internalization pathway. Cells with concentration 1 × 10^4^ cells per ml in a 96 well plate (tissue culture grade). In 100 ml of culture medium consist of DMEM medium containing 10% FBS and 1% antibiotic mixture, The MCF-7 cells were seeded at a density of 1 × 10^4^ per 100 ml DMEM into 96-well plates. MCF-7 cell line is used to follow DOX path in the tumor cell. Cs/DOX-MNPs (500 μg/ml) is incubated for 24 h in two Petri dishes. The cells treated with Cs/DOX-MNPs were washed three times with PBS. An external magnet is put under one dish and the other dish without magnet. The cellular internalization of nanoparticles was observed by fluorescence microscopy after adding 4′,6-diamidino-2-phenylindole (DAPI) stain for cell nuclei staining [28, 29].

Magnetically guided drug delivery involves an external magnetic field to deliver nanoparticles to a desired target area where the medication is needed [30, 31].

### Cytotoxicity assay-MTT

The cytotoxicity studies through MTT test with and without external magnetic field for the free DOX and the Cs/DOX-Cit-MNPs towards human tumor breast cell (MCF-7) and the normal human cell (WiSH) lines were carried out according to method previously described [32–35].

#### Culture media

Dulbecco Modified Eagles Medium (DMEM) containing 2 m Ml-glutamine, 100 Units/ml penicillin and100 g/ml streptomycin supplemented with 5% foetal bovine serum (complete media).

#### Preparation of cell suspension for the assay

The desired human cancer cell lines, MCF-7 (breast cancer cell line) and the normal human cell (WiSH) lines were grown at 37 °C, 5% CO_2_ and 90% relative humidity till sub-confluent stage. The cells were then harvested by treatment with trypsin EDTA solution. The number of cells was counted in a haemocytometer and the cell density was adjusted to75,000 cells/ml in complete media.

MTT assay was carried out in triplicates in 96 well microtiter culture plates. 100 µl of the cell suspension (7500 cells) was added into each well of the 96 well plates and incubated at 37 °C, 5% CO_2_ and 90% relative humidity for 24 h.

After 24 h, cells were treated with different concentrations (6.5, 12.5, 25 and 50 µg) of DOX alone, (6.5, 12.5, 25, and 50 µg) DOX loaded nanoparticles and the control group is formed of free cells. The plates were incubated for a further period of 24 h in the CO_2_ incubator. 20 µl of 5 mg/ml MTT was added into each well and the plates were incubated for 3.5 h. At the end of incubation period, culture media was carefully removed and 150 μl MTT solvent was added into each well. After covering the plates within foil, the plates were agitated on orbital shaker for 15 min. Absorbance was read at 590 nm.

The average values from triplicate readings were determined [35] and the average value for the blank was subtracted and the inhibition rate %, was calculated from the following equation:

2 $${\text{Cell inhibition rate }}\left( {{\text{IR }}\% } \right) \, = { 1}00 \, - \, \left\{ {\left( {{\text{As}} - {\text{Ab}}} \right)/ \, \left( {{\text{Ac}} - {\text{Ab}}} \right)} \right\} \times {1}00,$$

where, As = Absorbance value of sample. Ab = Absorbance value of blank. Ac = Absorbance value of control.

## Results and discussions

### Preparation and reaction mechanism of nanoparticles

#### Preparation and reaction mechanism of naked Fe_3_O_4_ nanoparticles (MNPs)

The MNPs were prepared by co-precipitation of Fe^2+^ and Fe^3+^ ions in an alkaline media. Ferrous to ferric chloride was poured drop-wisely to sodium hydroxide, under vigorous stirring and N_2_ flow. The Magnetite is formed immediately and removed from solution through an external magnetic field.

During the precipitation of MNPs from Fe^2+^ and Fe^3+^ salts mixture, two separate reactions may be occur after adding sodium hydroxide till the black MNPs are observed [36].

The possible reaction for the formation of MNPs is as follows:

$$\begin{array}{l} {\text{Fe}}^{{{3} + }} + {\text{ 3OH}}^{ - } \to {\text{ Fe}}\left( {{\text{OH}}} \right)_{{3}} \hfill \\ {\text{Fe}}\left( {{\text{OH}}} \right)_{{3}} \to {\text{ FeO}}\left( {{\text{OH}}} \right) \, + {\text{ H}}_{{2}} {\text{O}} \hfill \\ {\text{Fe}}^{{{3} + }} + {\text{ 2OH}}^{ - } \to {\text{ Fe}}\left( {{\text{OH}}} \right)_{{2}} \hfill \\ {\text{2 FeO}}\left( {\text{OH }} \right) \, + {\text{ Fe}}\left( {{\text{OH}}} \right)_{{2}} \to {\text{ Fe}}_{{3}} {\text{O}}_{{4}} \downarrow \, + {\text{ 2H}}_{{2}} {\text{O}}{.} \hfill \\ \end{array}$$

### Reaction mechanism of Cit-MNPs

**Fig. 1:**
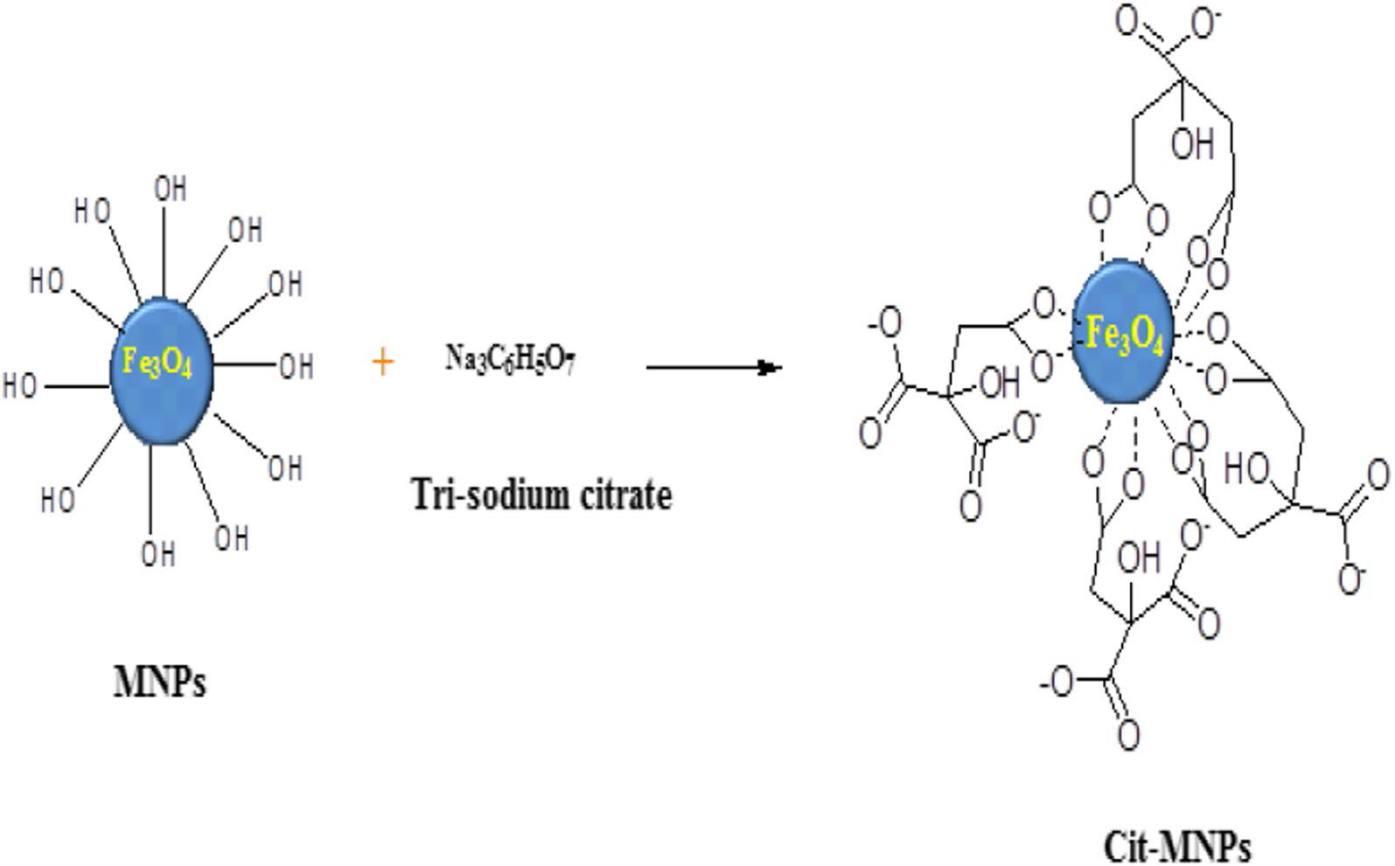
Reaction mechanism of Fe_3_O_4_ nanoparticles with tri-sodium citrate

Two reactions were involved in this process. First, the tri-sodium citrate was hydrolyzed to highly reactive carboxylic groups in alkaline medium. Then, they condensate with free –OH groups on the surface of magnetite to form Fe–COOH as shown in Fig. 1 [4].

#### Reaction mechanism of DOX/Cit-MNPs

**Fig. 2:**
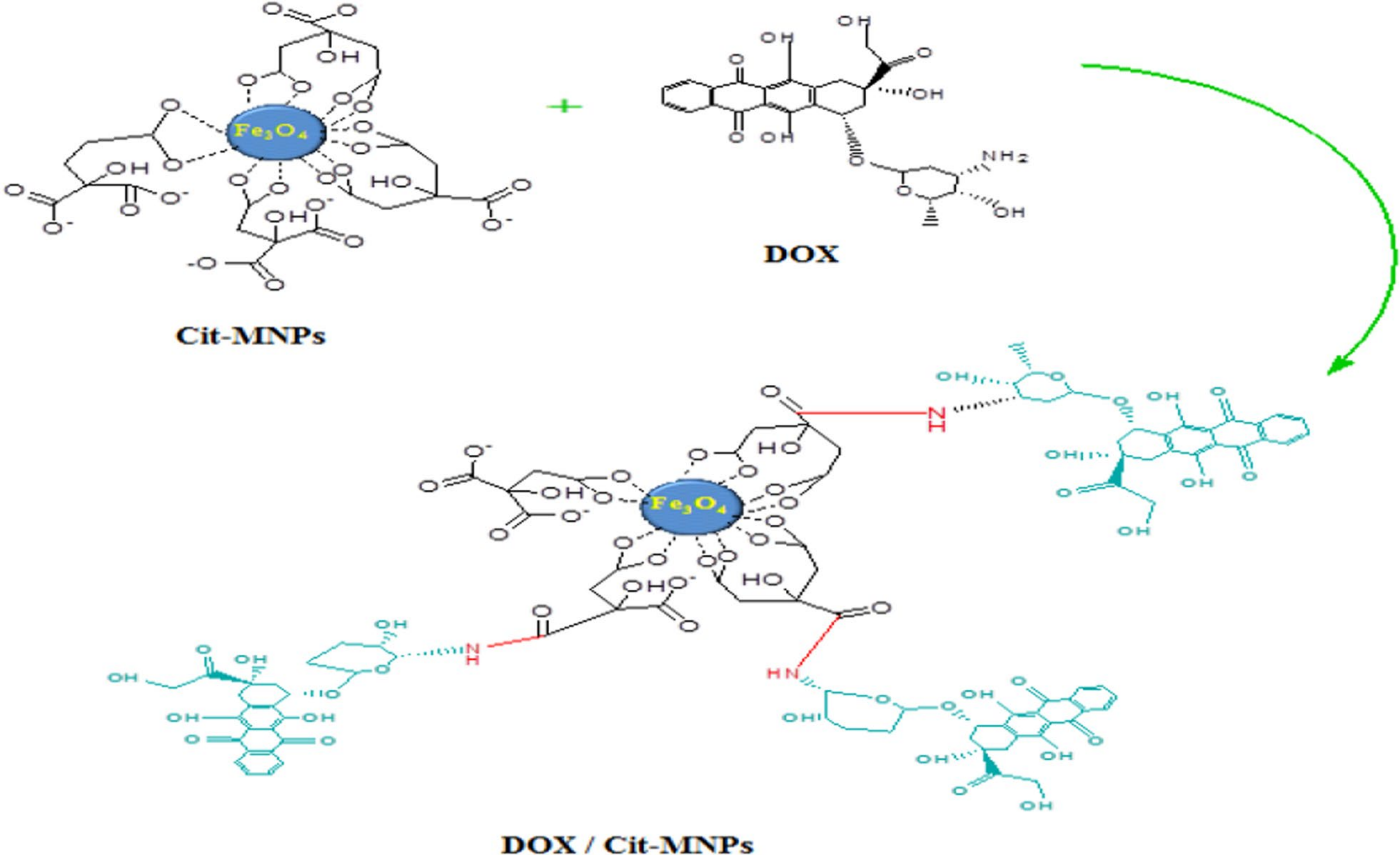
Reaction mechanism of DOX with Cit-MNPs

DOX was conjugated directly with Cit-MNPs through the reaction between carboxylic group of Cit-MNPs and amine group of the DOX via Schiff base chemistry. The reaction mechanism is schematically represented in Fig. 2.

#### Reaction mechanism of Cs/DOX/Cit-MNPs

**Fig. 3:**
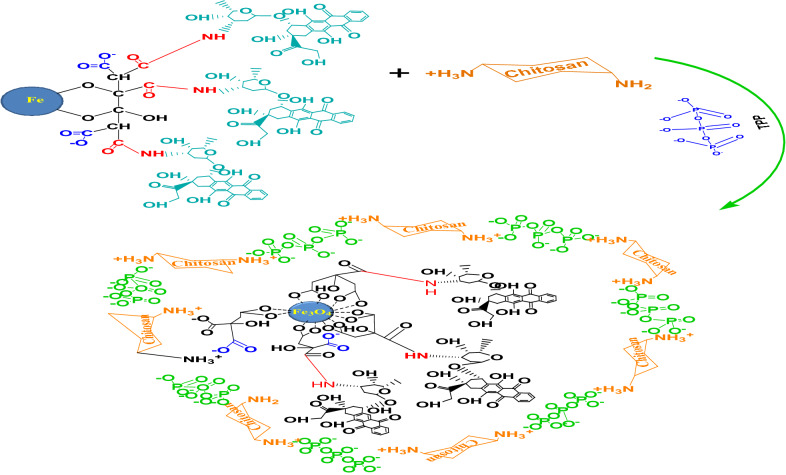
Reaction mechanism of chitosan with DOX/Cit-MNPs

To overcome the side effects of DOX, it was coated with chitosan as a natural biocompatible and smart biodegradable polycation [37]. The coating process occur through cross linking method by using STPP solution as an ionic cross linker between chitosan layers. The reaction mechanism is schematically represented in Fig. 3.

### Characterization

#### FTIR spectra

The FTIR spectra of MNPs, (Additional file 1: Fig. S1a), confirmed the formation of MNPs as the Fe–O characteristic peaks appear at 573 cm^−1^. In aqueous medium, the surface of the MNPs has been surrounded by OH groups that absorb at about 1623 cm^−1^ (deforming) and 3409 cm^−1^ (stretching).

The FTIR spectra of Cit/MNPs, (Additional file 1: Fig. S1b) indicated the reaction between MNPs and tri-sodium citrate as a strong band appears at 586 cm^−1^ which is the Fe–O characteristic peaks of MNPs. The vibrations of asymmetry and symmetry stretching from the coordinated carboxyl anion of tri-sodium citrate were located at 1625 and 1446 cm^−1^, respectively. The absorbance peak at 3434 cm^−1^ represents stretching band of O–H groups surrounding the surface of MNPs and band at 1625 cm^−1^ because of complexing H_2_O with Cit-MNPs.

The FTIR spectrum, (Additional file 1: Fig. S1c) demonstrated that DOX is conjugated with Cit-MNPs through imine bond between carboxylic groups on the surface of Cit-MNPs and amine group of DOX. This imine bond appears at 1033 cm^−1^; the band that appears at 1790 cm^−1^ refers to the carboxylic group on DOX. The Vibrational bands appearing at 1627 and 1424 cm^−1^ of C=O and C–O stretching vibrations refer to some carboxylic groups that are not conjugated with DOX. The Fe–O characteristic peak of MNPs appears at about 576 cm^−1^.

The FTIR spectra for Cs/DOX/Cit-MNPs, (Additional file 1: Fig. S1d), prove that DOX/Cit-MNPs are successfully coated with chitosan layer as N–H stretching vibration overlaps with OH stretching at 3417 cm^−1^ and the 1620 cm^−1^ peak of N–H bending vibration; the vibrational band at 1161 cm^−1^ refers to (P–O) of STPP which proves the crosslinking between chitosan and MNPs–COOH loaded with DOX. A new sharp peak at 576 cm^−1^ related to Fe–O group, specific for the stretching vibration of Fe–O groups from the magnetite nanoparticles, confirmed the loading of the Fe_3_O_4_ on chitosan [38–40].

The FT-IR spectra show typical vibration bands of the MNPs, Cit-MNPs, DOX/Cit-MNPs and Cs/DOX/Cit-MNPs according to other studies [14, 41, 42]. Due to the fact that DOX is rich in amine groups, the loading step was confirmed by the peaks attributed to the imine bonds. This implies that the synthesized Cs/DOX/Cit-MNPs have properly been loaded with DOX [14, 43].

#### Zeta potential

The surface charges of naked MNPs, Cit-MNPs, DOX/Cit- MNPs and the Cs/DOX l/Cit-MNPs are reported using zeta potential. All samples were suspended in water and 10^−3^ M NaCl aqueous solutions at pH 4–9 (adjusted by NaOH or HCl) with concentration (1 mg/ml).

The zeta potential of the naked MNPs, Cit-MNPs, and the Cs/DOX/Cit- MNPs in deionized water were 32.2 ± 4, − 30.3 ± 5 and 40.6 ± 3 mV, respectively.

For Cit-MNPs converting the net charge of MNPs to negative charge (− 30.3 ± 5 mV) in water with good colloidal stability because of the effect of citrate ions.

**Fig. 4:**
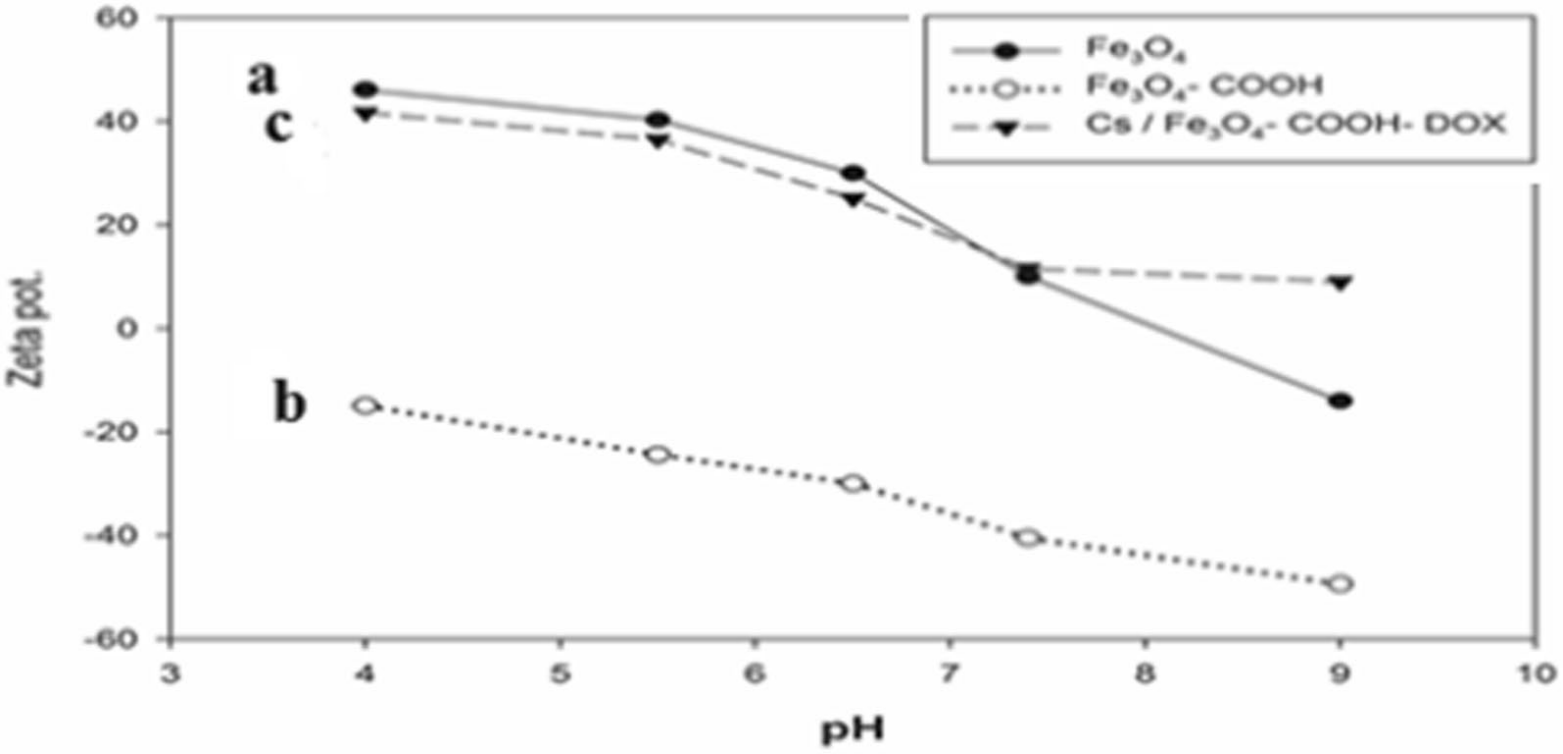
Relation between zeta potential and different pHs of (**a**) Fe_3_O_4_ magnetite, (**b**) Cit-MNPs and (**c**) chitosan coated DOX-Cit-MNPs

The Cs/DOX/Cit-MNPs are positively charged with 40.6 ± 3 m.v which means that chitosan forms a positively shell around the DOX/Cit- MNPs that will increase the cellular internalization by the cancer cells than negatively charged particles. The effect of different pHs on the zeta potential of nanoparticles shown in Fig. 4 and it proves that the surface charge of both nanoparticles are very sensitive to pH of surrounding media.

#### TEM and SEM analysis

**Fig. 5:**
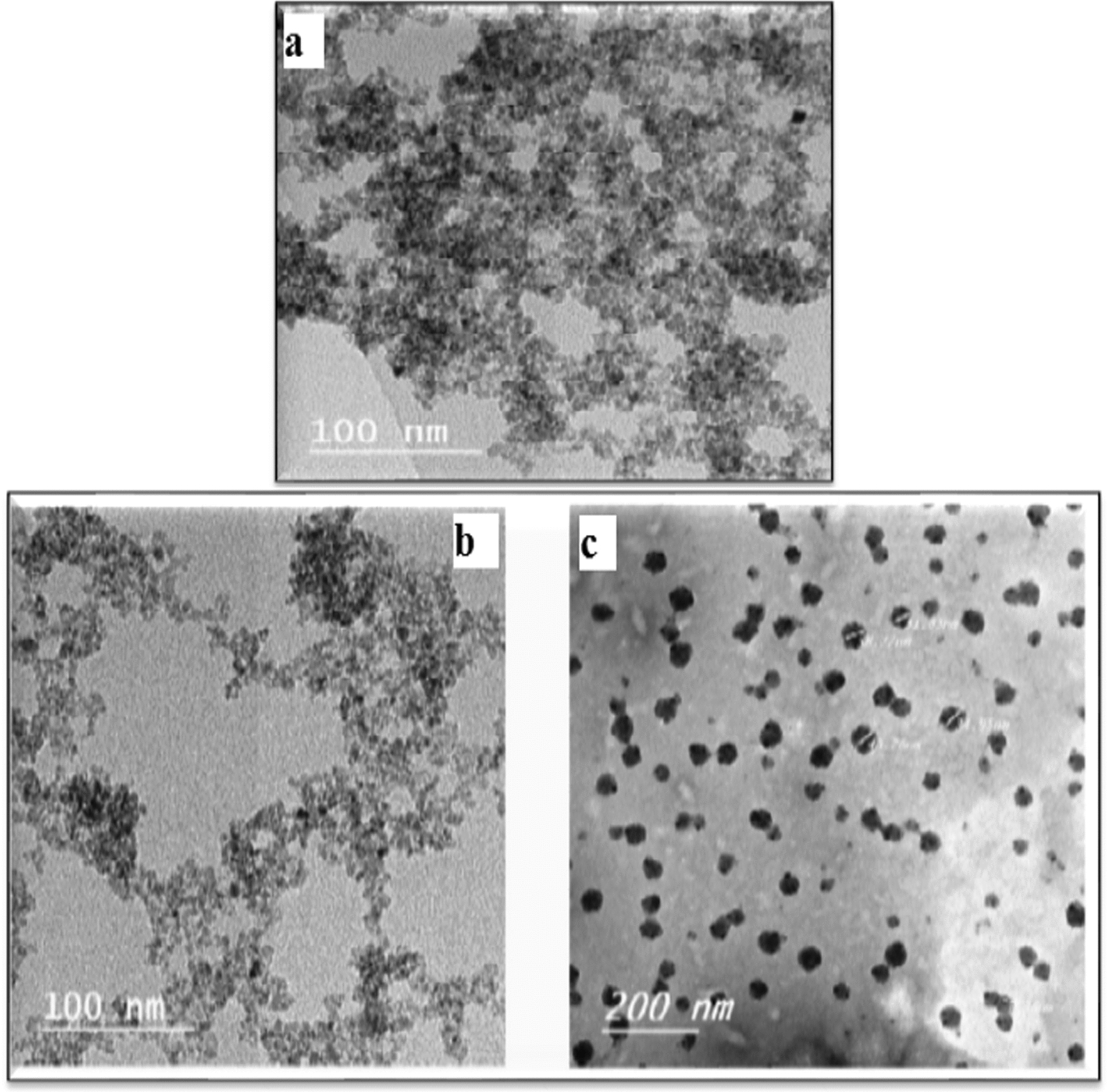
TEM images of (**a**) naked MNPs (**b**) Cit-MNPs and (**c**) Cs/DOX/Cit-MNPs

The TEM results reported in Fig. 5a, b indicated that naked MNPs and Cit-MNPs are successfully synthesized in nanoscale with diameter ranged from 3.5–7.8 nm and 5–8 nm, respectively with uniform spherical shape and good disparity. The Cs/DOX Cit-MNPs were also formed with spherical shape with diameter ranged from 40–55 nm and with good disparity (Fig. 5c).

The surface morphology of naked MNPs, Cit- MNPs and Cs/DOX Cit-MNPs was observed from SEM images in (Additional file 1: Fig. S2a–c). The spherical shape and the homogeneity structure of nanoparticles for naked MNPs, Cit- MNPs and Cs/DOX Cit-MNPs, can be noticed.

The morphological studies of the present work by SEMand TEMhave been spherically. Morphological studies by SEMof BPPE-CCMNPs revealed that the MNPs have been spherically formed. However, TEMimages taken from BPPE-CCMNPs display mostly the core of the nanocomposite [14]. This could be due to the collapse of the chitosan layer on the surface of the magnetic core on sample dispersion prior to the imaging step [14, 44]. Nevertheless, images taken from the synthesized BPPE-CCMNPs show that they are roughly spherical in shape [14]. Images achieved from AFM were on par with SEMand TEMresults, but showed a bit of aggregation [14].

#### EDX analysis

**Fig. 6:**
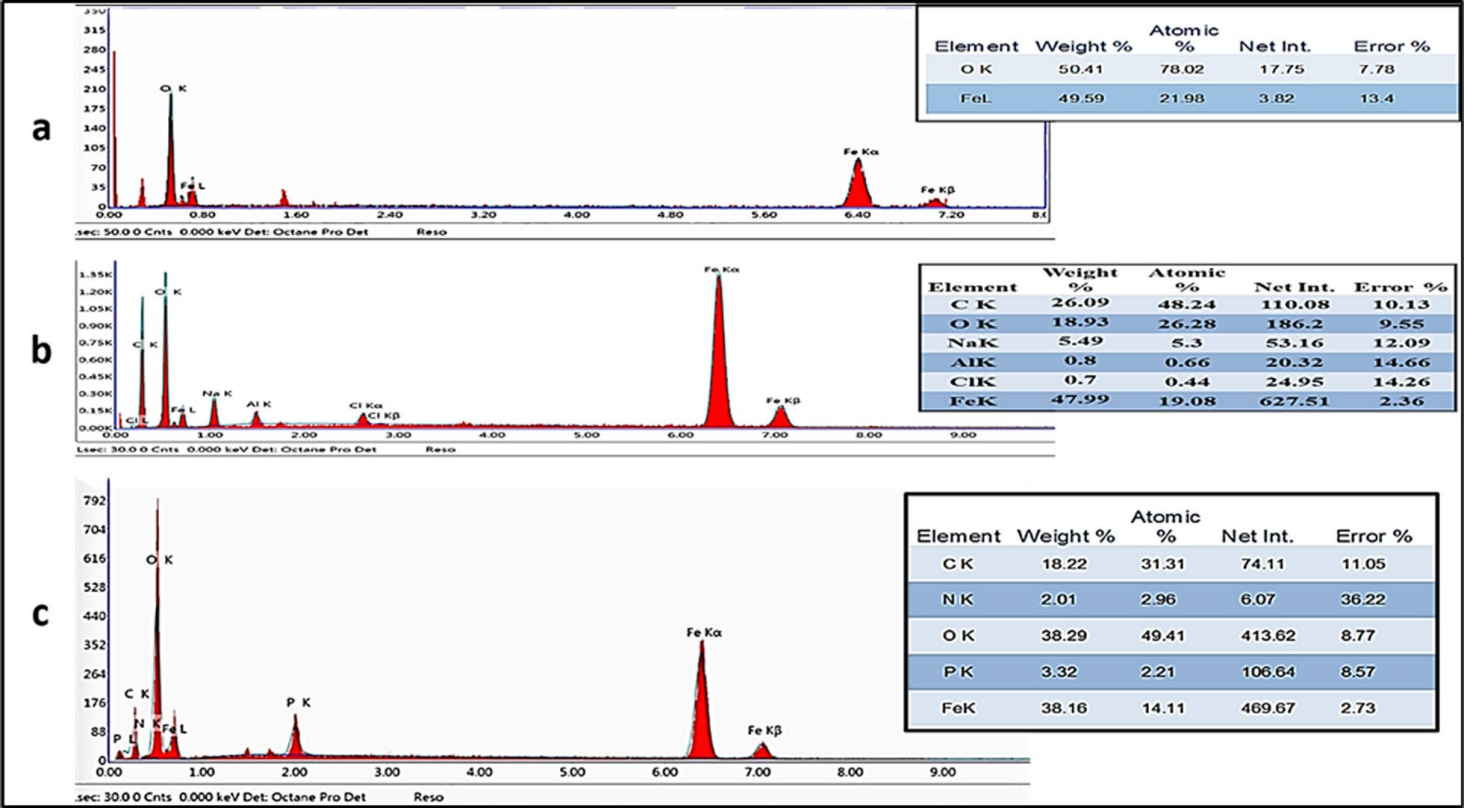
EDX analysis for a) naked MNPs, b) citrate magnetite nanoparticles (Cit-MNPs) & c) chitosan/DOX-citrate magnetite nanoparticles (Cs/DOX/Cit-MNPs)

To determine the elemental composition of the samples, SEM/EDS was used. From the results obtained in Fig. 6a for naked Fe_3_O_4,_ the spectrum contained two peaks, which were assigned to Fe and O.

The Cit-Fe_3_O_4_ in Fig. 6b contains 3 main peaks that refer to Fe, O and C with traces of Na. The presence of carbon peak with 19.55 weight % indicate the functionalization with citrate ions with traces of Na may be due to slight amount of sodium citrate that are not washed well. The Cs/DOX Cit-MNPs in Fig. 6c have 5 main peaks that refer to Fe, O, C, N and P. The presence of C and N prove the functionalization of iron oxide surface with sodium citrate. The presence of N peak proves the coating with chitosan to reach 2.1%. The presence of P peak with 3.3% may be from the STPP used to crosslink the chitosan layer with each other and with iron oxide nanoparticles.

#### XRD analysis

**Fig. 7:**
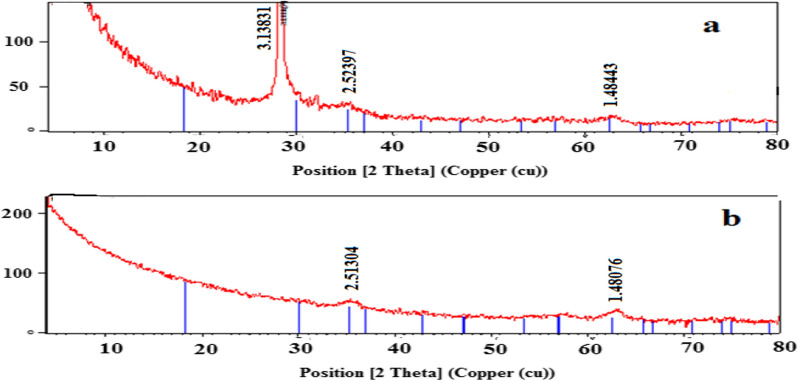
XRD spectra for (**a**) Cit-MNPs and (**b**) Cs/DOX-Cit-MNPs

XRD analysis confirms the crystalline properties of the Cit-MNPs, Cs/DOX Cit-MNPs and confirmed the presence of pure phase of magnetite. Representative powder XRD patterns of two samples are presented in Fig. 7a, b. The presence of sharp and intense peaks proves the formation of highly crystalline nanoparticles. According to the literature, iron oxide nanoparticles show sharp prominent peaks at 30.5 (220), 35.84 (311), 43.46 (400), 53.90 (422), 57.38 (511) and 62.90 (440) with 311 peak having the highest intensity [45–48] in agreement with ICCD magnetite card number 00-019-0629. These peaks with similar intensities and with the corresponding angles were present in all of the tested samples (Fig. 7a). This indicates that the used synthesis technique for Fe_3_O_4_ was feasible [46, 47, 49, 50].

It was also noted from Fig. 7b that the addition of chitosan did not affect the crystalline structure of Cit-MNPs. The average particle size of MNPs can be calculated using Scherer’s equation (Eq. 3).

3 $${\text{Scherer equation D}} = {\text{K}}\lambda /\left( {\beta {\text{cos}}\theta } \right),$$

where, K is a constant, λ is X-ray wavelength, β is the peak width of half-maximum and θ is the Bragg diffraction angle [51]. The diameter of MNPs was calculated by Scherer’s equation to be 6 nm for Cit- MNPs and 35 nm for Cs/DOX/Cit-MNPs.

The synthesis of Cit-MNPs, Cs/DOX Cit-MNPs was confirmed by XRD. It could be determined from the diffraction peaks of the Cit-MNPs, Cs/DOX/Cit-MNPs that the synthesized MNPs have sustained their purity after surface modification and drug loading step [14, 42].

#### Thermogravimetric analysis

The TGA curves of Cs/DOX/Cit-MNPs are shown in (Additional file 1: Fig. S3). Three weight losses are observed in the TGA diagram. The initial weight loss about 9% may be due to the evaporation of absorbed H_2_O between 50 and 156 °C. The weight loss of about 4% in the temperature range 150–600 °C is due to the decomposition of Cs layer.

#### Magnetic properties of naked and Cs/DOX/Cit- MNPs

The hysteresis loop in the magnetization curve presented in (Additional file 1: Fig. S4) illustrates that, the remanence (residue magnetization) and coercive force (the applied field that reduces magnetization to zero) were zero and there was no magnetic hysteresis loop observed, proving the characteristic superparamagnetic behavior of all prepared MNPs. The saturation magnetization (Ms) values were found to be 60 emu/g for naked (Additional file 1: Fig. S4a) and 55 emu/g for Cit-MNPs (Additional file 1: Fig. S4). This difference in magnetization values (Ms) between naked MNPs and Cit-MNPs may be due to the presence of sodium citrate on the surface of magnetite nanoparticles as a denser coating [52]. This effect of sodium citrate on the saturation magnetization (Ms) value is negligible in comparison with the other coating materials as silica (34.3 emu/g) [53]. On the other hand, the saturation magnetization (Ms) values for Cs/DOX/Cit-MNPs were found to be 48 emu/g (Additional file 1: Fig. S4c).

Assessments of the magnetic properties of the nanoparticles show that the MNPs have satisfactory magnetic potential. However, at the coating and drug loading step, the magnetic potential has gradually been decreased. The magnetic property reduction of the nanoparticles at these steps could potentially be due to the addition of the new layers which hinders the magnetic strength of the MNPs [42]. Owing to the magnetic potential of the synthesized Cs/DOX/Cit-MNPs, it could be used as targeted therapy by external magnetic field in breast cancer drug delivery for further studies [14, 54].

### DOX loading efficiency %

The loading efficiency % increases with increasing DOX concentration to reach 99.6% for 30% DOX (w %), Table 1. The high loading % may be due the large surface area and large number of active carboxylic groups that interact with these different concentrations from DOX to reach 99.6% for 30% (w %) from Cit-MNPs.

**Table 1:** Loading efficiency % of DOX-Cit-MNPs with different DOX concentrations

Sample	Drug concentrations (%)	Loading efficiency (%)
DOX-Cit-MNPs	10	98.2
	20	99
	30	99.6

Studies on drug loading of the Cit-MNPs show demonstrated that the loading efficiency % increases with increasing DOX concentration. The high loading % may be due the large surface area and large number of active carboxylic groups that interact with these different concentrations from DOX to reach 99.6%. Taherian et al. [14] studied drug loading of the Chitosan-coated magnetic nanoparticles (CCMNPs) show rapid loading of Black pomegranate peel extract (BPEE) at the first stages [14]. However, when time passed, the absorption rate was moderately decreased. Since there was not much change in loading efficiency of the CCMNPs from 120 to 180 min, it could accordingly be concluded that the CCMNPs have reached their maximum loading capacity. This could be due to the incorporation of the polyphenols into the chitosan layer [42].

### In-vitro release studies of DOX from Cs/DOX/Cit-MNPs

**Fig. 8:**
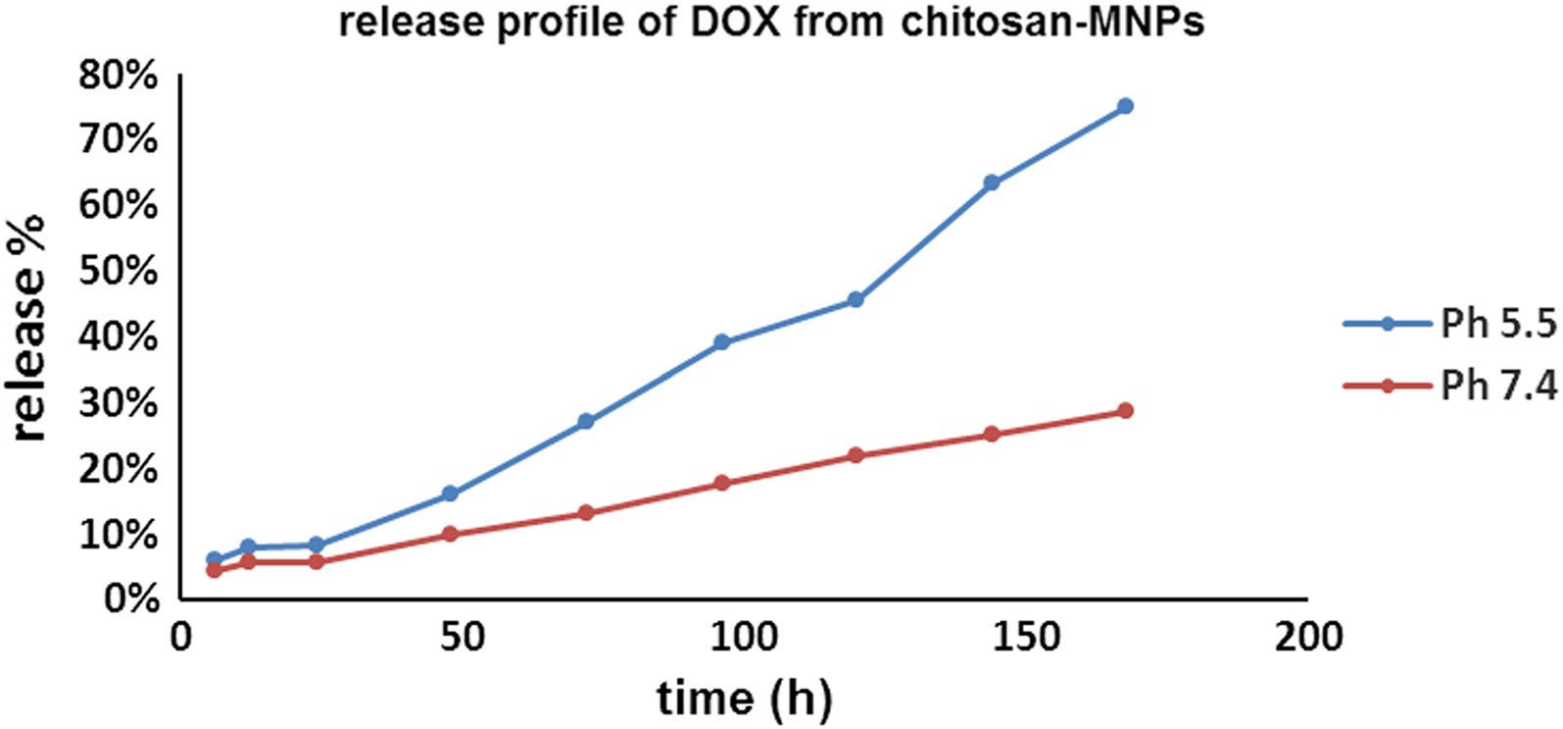
Release profile of DOX from chitosan-Cit-MNPs nanoparticles at (**a**) pH 5.5 and (**b**) pH 7.4

The drug release behavior of DOX from Cs/DOX/Cit-MNPs was studied in the physiological pH (7.4) and in acidic media at pH 5.5. From the results shown in Fig. 8, the Cs/DOX/Cit-MNPs offer a sustained DOX release from core shell nanoparticles in acidic media at pH 5.5, Fig. 8a. A burst release occurred in the first 6 h to reach 6%, then a sustained release began to be 12% after 24 h to reach 75% after 168 h.

At pH 7.4, Fig. 8b, an initial burst release occurred in first 6 h to reach 4.3%, then sustained release began to reach 7% after 24 h and followed by controlled sustained release to reach 28.6% after 168 h.

The obtained results indicated the pH-dependency in drug release rate from the nanocarriers as it depends on the cleavage of acid-labile linker used for drug conjugation to the MNPs nanoparticles. Our results are consistent with the other researcher’s report [55] which demonstrates that the weak acidic condition could accelerate cleavage of the imine bonds linker.

### Cellular internalization of Cs/DOX-Cit-MNPs

**Fig. 9:**
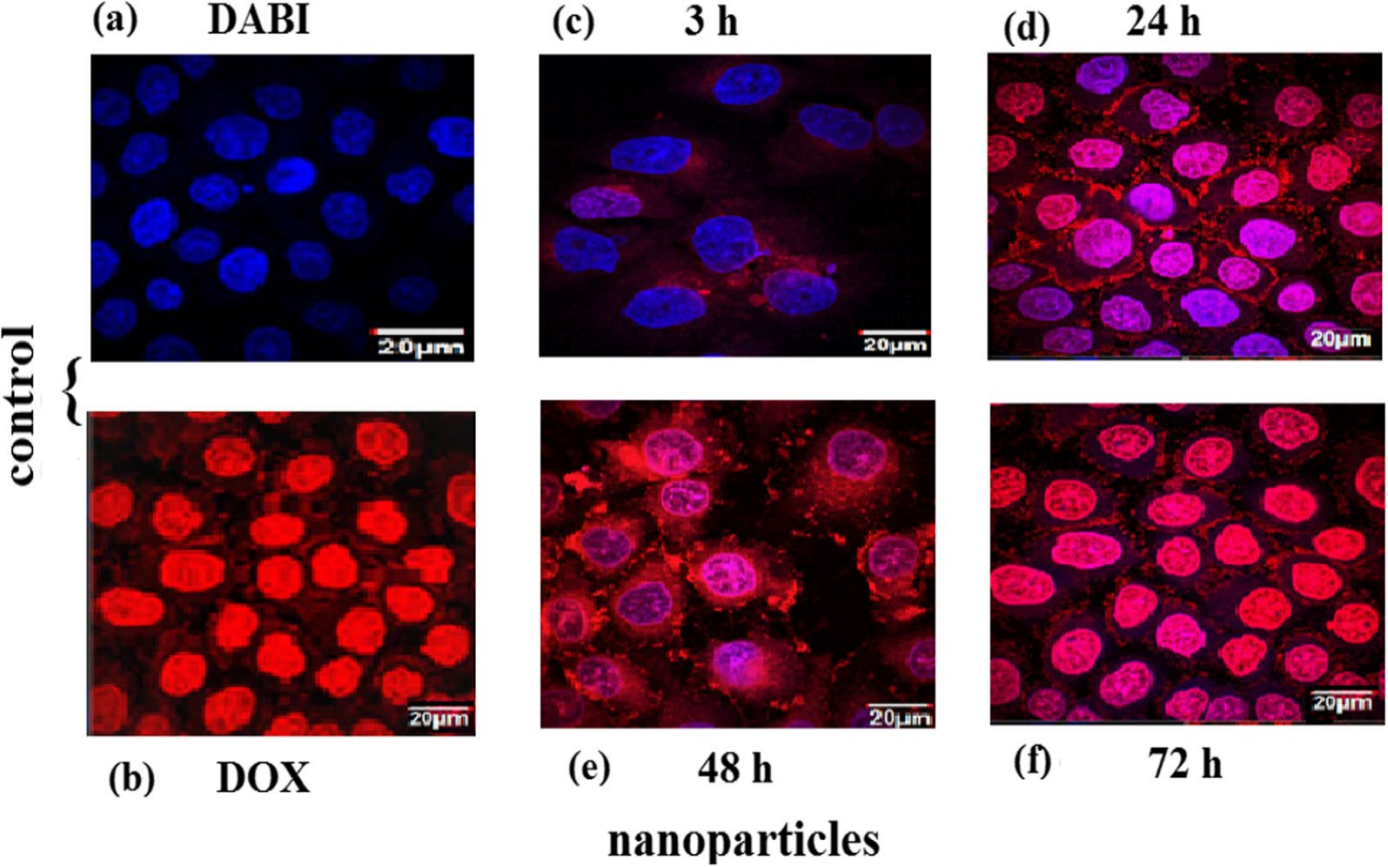
Fluorescent microscopy images without external magnet. **a** Control of DAPI stain labelled nuclei of the cells. **b** Control of free Doxorubicin, (**c**–**f**) fluorescence of internalized Cs/DOX-Cit-MNPs inside the MCF-7 cells after 3, 24, 48, 72 h, respectively

**Fig. 10:**
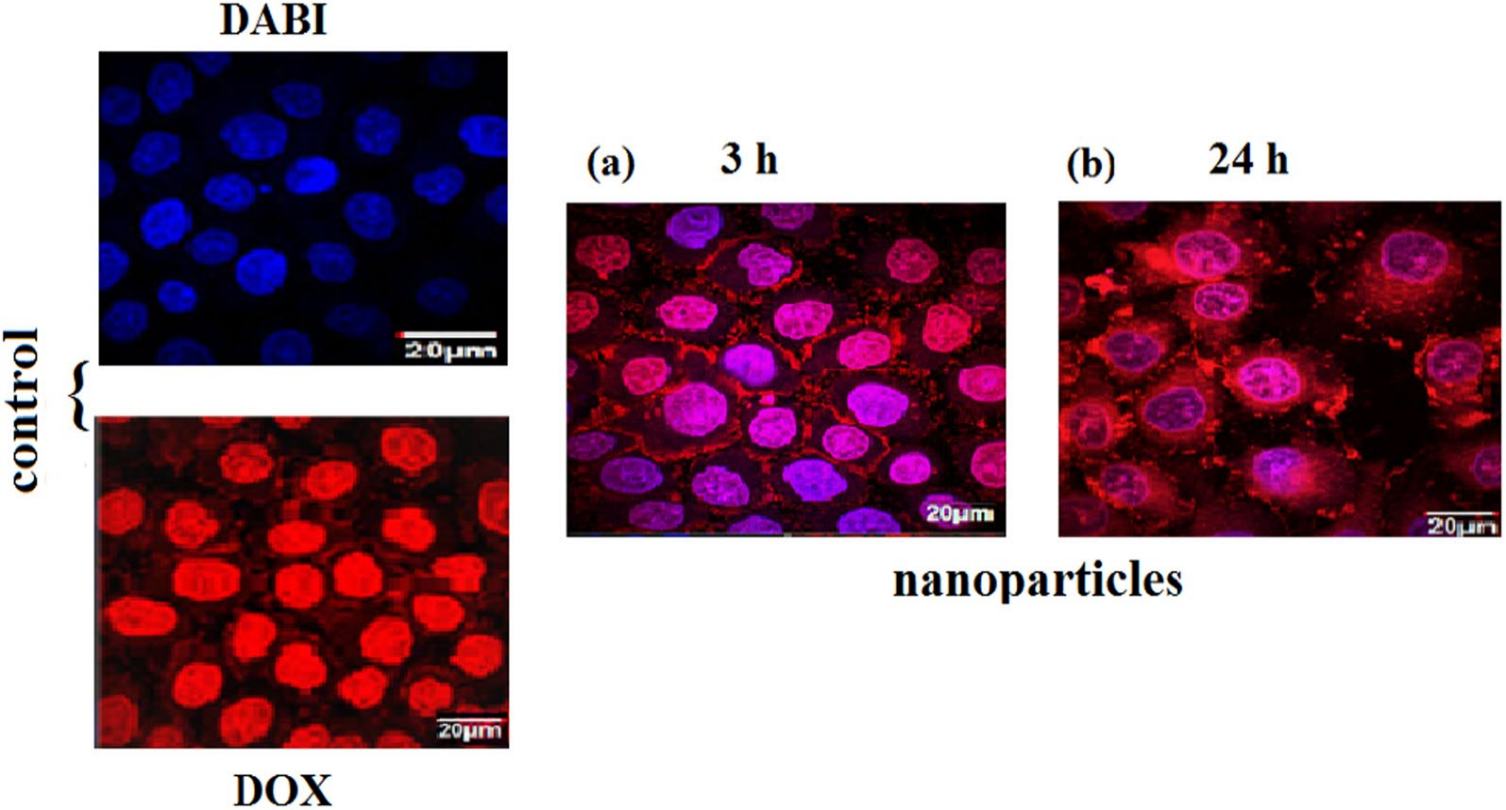
Fluorescent microscopy images with external magnet. **a**, **b** Fluorescence of internalized Cs/DOX-Cit-MNPs inside the MCF-7 cells after 3, 24 h, respectively

The internalization of Cs/DOX-Cit-MNPs on MCF-7 cells was examined by fluorescence microscopy with Fig. 9 and without external magnet, Fig. 10. DOX itself is a fluorescent compound detected with red filter of fluorescence microscope that appears with red color. The control images of DAPI staining and free DOX are presented in Fig. 9a, b. In Fig. 9c–f, the red color proves that Cs/DOX-Cit-MNPs were internalized to the cells, and the deepness of red color refer to the concentrations of nanoparticles in the cells compared to the control images of DAPI staining and free DOX.

The internalization mechanisms of free DOX and Cs/DOX-Cit-MNPs are different. As free DOX internalized the tumor cell by diffusing through its cell membrane but Cs/DOX-Cit-MNPs are internalized by endocytosis mechanism [56, 57]. This endocytic path involved three stages with three different pHs. Firstly, the early-endosomes with pH near 7.4, followed by endo-lysosomes with pH 5.5–6.0 and be around 4.5 in lysosomes.

By using an external magnet for targeting the nanoparticles to the MCF-7 cells, we observed increasing in red color on the cells which refer to nanoparticles accumulation inside the tumor cell due to the magnetic targeting as shown in Fig. 10.

So, Cs/DOX/Cit-MNPs will offer a responsivity to pH and selectively drug release in the malignant cells of the targeted organ with magnetically targeting responsivity.

### Cytotoxicity studies of Cs/DOX/Cit-MNPs

The cytotoxic effects of Cs/DOX-Cit-MNPs to breast cancer cells using the in vitro MTT assay without external magnet are illustrated in Table 2. With increasing DOX concentration and treatment time, the IR % of DOX solution and Cs/DOX-Cit-MNPs suspension to all cells is increased. Cs/DOX-Cit-MNPs showed sustained release and good inhibition to cancer cells.

**Table 2:** show the comparison between inhibition rate (IR %) of Cs/DOX-Cit-MNPs with different concentrations (6.5, 12.5, 25, 50 μg/ml) after 24, 48 and 72 h toward MCF-7 & WISH

Sample	Cells	IR, %											
		After 24 h				After 48 h				After 72 h			
		Drug concentration (mg)											
		6.5	12.5	25	50	6.5	12.5	25	50	6.5	12.5	25	50
Cs/DOX-Cit-MNPs	Tumor cells (MCF-7)	15	24	33	40	25	38	45	60	31	45	60	76
	Normal cell (WISH)	3	6	10	12	5	5	12	17	5	8	13	22
Free DOX	Tumor cells (MCF-7)	20	31	50	75	24	46	72	88	25	46	75	91
	Normal cell (WISH)	12	25	36	59	18	36	55	72	18	38	60	76

All results are expressed as the mean ± standard deviation with (n) = 6 and p < 0.001

As shown in Table 2, after 24 h, the inhibitory activity of Cs/DOX-Cit-MNPs suspension toward MCF-7 & WiSH cell lines was lower than that of free DOX solution for all concentrations to be 75 ± 0.06%, 59 ± 0.002 for free DOX and 40 ± 0.03%, 12 ± 0.01% for Cs/DOX-Cit-MNPs at the highest conc. (50 μg/ml) (P > 0.001).

After 48 h Table 2, the IR % of Cs/DOx-Cit-MNPs and DOX solution toward MCF-7 cell line were 60 ± 0.09% for Cs/DOX-Cit-MNPs and 88 ± 0.09% for free DOX at the highest conc. (50 μg/ml) (P < 0.001). A very significant difference was observed in IR% after 48 h between the free DOX & Cs/DOX-Cit-MNPs toward normal cell line (WiSH) to be 72 ± 0.05% and 17 ± 0.003, respectively.

After 72 h Table 2, IR% was 93 ± 0.07% for free DOX and 76 ± 0.10% for Cs/DOX-Cit-MNPs toward MCF-7 cell line. The same significant difference in IR% between free DOX & Cs/DOX-Cit-MNPs toward normal cell line (WiSH) was observed to be 76 ± 0.04% and 22 ± 0.07%, respectively.

From these results, Cs/DOX-Cit-MNPs offer a pH-sensitive sustained release for DOX from the MNPs through the chitosan shell to the outer media which explain the low IR % in the first 24 h then the IR %, increased by time to reach the maximum after 72 h with IR% = 76 ± 0.10.

The other important feature gained from Cs/DOX/Cit-MNPs nano carrier is their non-toxicity toward normal cells (WISH).

The Cs/DOX-Cit-MNPs offer a protective mode for normal cells compared with free DOX with IR% 22 ± 0.07 after 72 h. This is may be due to the biocompatibility feature of chitosan and magnetite nanoparticles in addition to pH-sensitivity of chitosan which is another important factor in the low IR% toward normal cell as the pH of normal cell is approximately 7.4. At this pH chitosan shell shrinks and doesn't promote the DOX release from it.

When MTT assay was carried out by using an external magnet for Cs/DOX-Cit-MNPs toward MCF-7 cell line (pH = 5.5), it was found that the IR% highly increased in presence of external magnetic field to reach 71 ± 0.07% in the first 24 h and 98 ± 0.04% after 72 h in (Additional file 1: Fig. S5). This increase in IR% in the presence of external magnetic field is due to the super paramagnetic properties of magnetite nanoparticles which act as a remote control for the nanoparticles toward the tumor cells and targeted drug release in the desired place. Also chitosan shell plays an important role in this high IR% due to its positive charge and its mucoadhesivness properties which improve the cellular uptake and penetration of cell membrane.

#### Potential application of external magnet in real life

Magnetically guided drug delivery involves an external magnetic field to deliver nanoparticles to a desired target area where the medication is needed. The advantage being that the dosage of the medication can be reduced and the systemic effect of the drugs can be reach the minimum level. The superparamagnetic behavior implies that its magnetization disappears once the external magnetic field is removed [30, 31]. Based on these properties, the superparamagnetic nanoparticles could be transported through the vascular system, concentrated in a specific area with the aid of a magnetic field, with substances to assure their cellular internalization and activate the properties of magnetic drug targeting and release in specific organ.

Biological tissue is nearly ‘transparent’ to magnetic energy, and magnetic fields pass through tissue without being significantly absorbed or distorted by body tissues. Magnetic nanoparticles introduced into the body can therefore allow for high local energy delivery to the particles, compared to the less magnetic surrounding tissue. As a result, magnetic fields and magnetically responsive particles can be harnessed for therapy and drug delivery, particularly in cancer [61].

Two approaches are most common: (i) the use of magnetic particles to improve the accumulation of drugs in a desired region via magnetic targeting [59, 60]; and (ii) the use of magnetic fields to heat magnetic particles to directly induce hyperthermia in or ablation of diseased tissues [61] and/or to trigger the release of drugs from thermally-sensitive carriers [62, 63].

Magnetic drug delivery strategies rely on transferring externally applied magnetic energy to magnetic particles that have been introduced into the body [64]. One of the most commonly used magnetic particles for drug delivery are superparamagnetic iron oxide nanoparticles (SPIONs). Unlike ferromagnetic materials, in which materials are permanently magnetized, paramagnetic materials are magnetized only when placed into an applied field. When the magnetic field is removed, the moments revert to a random orientation [65].

Magnetic targeting, also referred to as magnetophoresis, has been proposed as a potential mechanism to improve the accumulation and penetration of magnetic drug carriers in tumors [58]. For magnetic targeting, drug and magnetic nanoparticles are encapsulated into a nanocarrier, and a strong external static magnet is used to accumulate the drug carrier at a target near the static magnet. For example, Marie et al. used a static external magnet to encourage magnetoliposome accumulation in glioblastoma tumors in mice [66], while Huang et al. have developed and tested doxorubicin loaded magnetic micelles in a squamous cell carcinoma model in rabbits [67]. Magnetic targeting has even been validated in humans. In fact, as early as 1996, Lübbe et al. showed that magnetic drug targeting could be used to concentrate epirubicin-conjugated nanoparticles in sarcomas in phase I and II clinical trials [68, 69].

Several types of iron oxide nanoparticles are US Food and Drug Administration (FDA)-approved for use in magnetic resonance imaging (MRI) as contrast agents that can improve image resolution and information content. New imaging modalities, such as magnetic particle imaging (MPI), directly detect magnetic nanoparticles within organisms, allowing for background-free imaging of magnetic particle transport and collection [30].

“Lab-on-a-chip” technology benefits from the increased control that magnetic nanoparticles provide over separation, leading to improved cellular separation. Magnetic separation is also becoming important in next generation immunoassays, in which particles are used to both increase sensitivity and enable multiple analyte detection.

More recently, the ability to manipulate material motion with external fields has been applied in magnetically actuated soft robotics that are designed for biomedical interventions.

Magneto thermal treatments have been approved in the European Union (EU), and they were also approved by the US Food and Drug Administration (FDA) in 2006 for phase I clinical trials in the treatment of prostate cancer.

Briefly, magneto thermal heating occurs when magnetic particles are subjected to alternating magnetic fields (AMFs). Through magnetic induction, nanoparticles in AFMs are selectively heated, providing for localized increases in temperature.

The programmable robots can generate metachronal waves, making them able to crawl and roll, depending on the strength of the magnetic field.

#### Comparison of the cytotoxicity potential of the Cs/DOX-Cit-MNPs and the results of recently published studies

The MTT assay studies of the present work show that synthesized Cs/DOX-Cit-MNPs are toxic against MCF-7 cell line cancerous cells without affecting normal cells.

Taherian et al. [14] studied cytotoxicity of Black pomegranate peel extract (BPPE) and Black pomegranate loaded chitosan-coated magnetic nanoparticles (BPPE-CCMNPs). They concluded that the MTT and LDH assay studies show that the free BPPE and BPPE-CCMNPs are toxic against MDA-MB-231 and 4T1 cancerous cells. The BPPE-CCMNPs show more cytotoxicity compared with free BPPE. Results show that BPPE-CCMNPs cause more significant cell viability reduction from 15 to 1000 µg/ml on both MDA-MB-231 and 4T1 cells at 24 h and 48 h incubation in comparison to free BPPE [14]. According to the studies on the pomegranate peel extract, the existing phenolic acids and flavonoids are presented in soluble-free, soluble esterified and insoluble-bound forms. Although it has been reported that the total free soluble phenolic and flavonoid compounds are higher than their insoluble-bound form, some of the hydroxybenzoic acid derivatives have insoluble bonds [69, 70]. The insoluble bonds could make it difficult for the BPPE to dissolve in cell media that leads to higher cell viability compared to BPPE-CCMNPs. On the other hand, CCMNPs cover the BPPE to avoid additional molecular reactions and increase the efficiency of the drug. It has been determined that the CCMNPs can have interaction with the negative domain of the cell membrane by electrostatic interactions owing to the positive zeta potential of the CCMNPs [71]. Furthermore, they are taken up by cells through endocytosis, in which the pathway begins at a pH of 7.4 and ends at pH 4.5 in lysosomes that most of the BPPE from CCMNPs would release [39].

In another study, the cytotoxicity studies on bortezomib as a highly water-insoluble drug in free form and loaded to CCMNPs also revealed that IC50 of the drug-loaded CCMNPs was much lower than the free drug which demonstrates the role of CCMNPs in drug efficiency improvement [72].

For instance, Song et al. have synthesized magnetic alginate chitosan NPs and used curcumin as a polyphenol-rich compound as a loading drug. Cellular uptake results on MDA-MB-231 and HDF cells have revealed that at the highest studied concentration, about 80% and 50% of curcumin and curcumin-loaded NPs are taken up by MDA-MB-231 and HDF cells, respectively [73]. The cytotoxicity studies showed that neither curcumin nor the drug-loaded NPs were toxic against HDF normal cells, but significant toxicity was observed in MDA-MB-231.

Comparing with nanocarrier-loaded standard chemotherapeutics, Rahimi et al. have synthesized dendric chitosan grafted polyethylene glycol MNPs and used doxorubicin and methotrexate as the loading agents. The combination of the two chemotherapeutic drugs loaded to NPs at 5 µg/ml caused MCF-7 cell viability reduction to about 30% after 48 h of incubation [74].

In another study, Vijayan et al. evaluated paclitaxel-loaded poly (lactic-*co*-glycolic acid) NPs on different MDA-MB cell series. At the concentration of 10 µg/ml, the cell viability of the MDA-MB-157, MDA-MB-231, MDA-MB-435, MDA-MB-436, and MDA-MB-468 incubated with paclitaxel-loaded NPs were 44.4 ± 1.2%, 34.6 ± 0.8%, 42.4 ± 1.4%, 35.3 ± 0.8%, 55.2 ± 0.8%, in that respect [75].

## Conclusion

In the present work, biocompatible and biodegradable Cs/DOX/Cit-MNPs nano carrier for DOX was successfully prepared which protect normal cells from the side effects of DOX and offer binary- targeting for tumor cells through magnetic targeting and pH sensitive release with better cellular uptake. The synthesized nano carriers are spherical particles with diameter that ranges between 7 and 45 nm. The crystalline structure for both was confirmed by XRD to be pure magnetite (Fe_3_O_4_). The magnetic properties of MNPs were investigated by (VSM) to be 60 emu/g for naked, 55 emu/g for Cit-Fe_3_O_4_ and 48 emu/g for Cs/DOX/Cit-MNPs. The in-vitro release of DOX from Cs/DOX/Cit-MNPs, was found to be pH dependent. The internalization mechanisms of free DOX and Cs/DOX-Cit-MNPs are different. As free DOX diffuses through the cell membrane but Cs/DOX-Cit-MNPs are internalized to the cells by endocytosis. The CS/DOX-Cit-MNPs offer a sustained release for DOX through the chitosan shell with low IR% in the first 24 h. Then it increased to reach 76 ± 0.10% after 72 h without magnetic directing. By using an external magnet the IR% of CS/DOX-Cit-MNPs increased to reach 71 ± 0.07% in the first 24 h and 98 ± 0.04% after 72 h. The MTT assay studies of the present work show that synthesized Cs/DOX-Cit-MNPs are toxic against MCF-7 cell line cancerous cells without affecting normal cells. The Cs/DOX/Cit-MNPs offer a very protective mode for normal cells compared to the free DOX.

## Supplementary Information

**Additional file 1:  Fig. S1**. IR spectra of (a) free magnetite, (b) Cit-MNPs, (c) DOX-Cit-MNPs, (d) chitosan / DOX-Cit-MNPs and (e) tri-sodium citrate.** Fig. S2**. SEM images of (a) naked MNPs,(b) Cit-MNPs and (c) chitosan coated DOX / Cit-MNPs.** Fig. S3**. TGA analysis for chitosan coated DOX-Cit-MNPs.** Fig. S4**. Magnetization curve of: (a) naked MNPs (b) Cit-MNPs, (c) chitosan coated DOX- Cit-MNPs and.** Fig. S5**. Comparison between inhibition rate (IR %) of Chitosan coated DOX-Cit-MNPs with different concentrations (6.5, 12.5, 25, 50 μg/mL) and free DOX with magnetic directing toward MCF-7 after 24, 48 & 72h by external magnet. The results are expressed as the mean ± standard deviation with (n) = 6 and p <0.001.

## Data Availability

All data generated or analysed during this study are included in this published article [and its Additional files].

## Abbreviations

STPP: Sodium tripolyphosphate
SPMNPs: Superparamagnetic magnetite nanoparticles
DOX: Doxorubicin hydrochloride
MCF7: Human breast cancer cell line
Cit-MNPs: Functionalized magnetite nanoparticles with tri-sodium citrate
DOX/Cit-MNPs: Tri-sodium citrate functionalized magnetite loaded DOX nanoparticles
Cs/DOX/Cit-MNPs: Chitosan coated tri-sodium citrate functionalized magnetite loaded DOX nanoparticles
WiSH: Human Normal cell line
NEDD: Nano-enabled drug delivery technique
MDT: Magnetic drug targeting
FDA: Food and Drug Administration
MRI: Magnetic resonance imaging
MNPs: Magnetite nanoparticles
COOH groups: Carboxylic groups
IR%: Inhibition rate %
L.E %: Loading efficiency %
Cs: Chitosan
DAPI: 4',6-Diamidino-2-phenylindole
MTT: 3-(4,5-Dimethylthiazol-2-yl)-2,5-diphenyltetr azolium bromide
TEM: Transmission Electron Microscopy
CCMNPs: Chitosan-coated magnetic nanoparticles
BPPE: Black pomegranate peel extract
BPPE-CCMNPs: Black pomegranate loaded chitosan-coated magnetic nanoparticles
RPMI: Roswell Park Memorial Institute
DMEM: Dulbecco’s modified Eagle medium
MTT: 2,2-Diphenyl-1-picrylhydrazyl
MNPs: 3-(4,5-Dimethylthiazol-2-Yl)-2,5-diphenyltetrazolium Bromide Magnetic nanoparticles
BP: Black pomegranate
BPP: Black peel pomegranate
