# Supplementary material for: Biodegradable functionalized magnetite nanoparticles as binary-targeting carrier for breast carcinoma
Source: BMC Chem. 2023 Feb 13;17(1):3. doi: 10.1186/s13065-023-00915-4 (PMC9926567; doi:10.1186/s13065-023-00915-4)
Supplement: Supplementary file 1 — Additional file 1: Fig. S1. IR spectra of (a) free magnetite, (b) Cit-MNPs, (c) DOX-Cit-MNPs, (d) chitosan / DOX-Cit-MNPs and (e) tri-sodium citrate. Fig. S2. SEM images of (a) naked MNPs,(b) Cit-MNPs and (c) chitosan coated DOX / Cit-MNPs. Fig. S3. TGA analysis for chitosan coated DOX-Cit-MNPs. Fig. S4. Magnetization curve of: (a) naked MNPs (b) Cit-MNPs, (c) chitosan coated DOX- Cit-MNPs and. Fig. S5. Comparison between inhibition rate (IR %) of Chitosan coated DOX-Cit-MNPs with different concentrations (6.5, 12.5, 25, 50 μg/mL) and free DOX with magnetic directing toward MCF-7 after 24, 48 & 72h by external magnet. The results are expressed as the mean ± standard deviation with (n) = 6 and p <0.001. [file 13065_2023_915_MOESM1_ESM.docx]

**Additional file 1**

**Biodegradable functionalized magnetite nanoparticles as binary-targeting carrier for breast carcinoma**

Magda Ali Akl* ^1^, Amira M Kamel^2^ and Mahmoud Ahmed Abd El-Ghaffar ^2^

^1^Chemistry Department, Faculty of Science, Mansoura University, Mansoura, Egypt

^2^Polymers and Pigments Department, National Research Centre, 33-El-Bohouth St. Dokki, Cairo, Egypt

***Corresponding author e mail: magdaakl@yahoo.com**


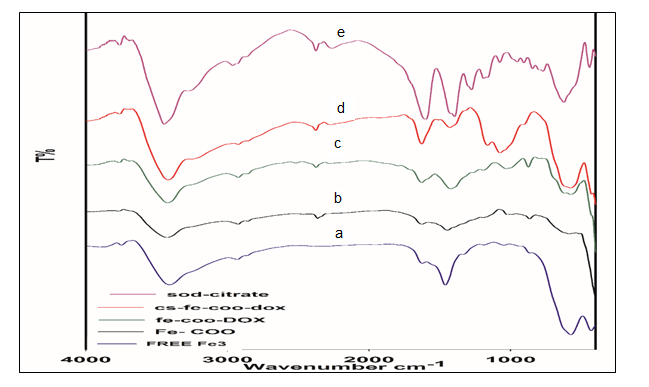
**Fig.S1. IR spectra of (a) free magnetite, (b) Cit-MNPs, (c) DOX-Cit-MNPs, (d) chitosan / DOX-Cit-MNPs and (e) tri-sodium citrate.**

***
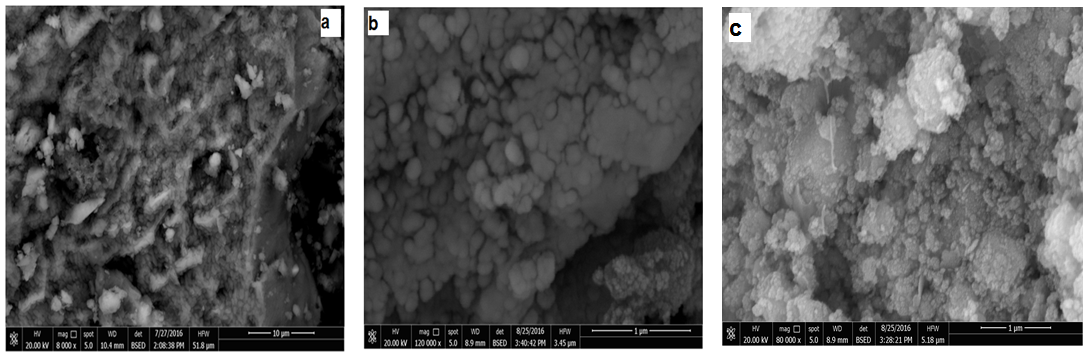
***

Fig.S2. SEM images of (a) naked MNPs,(b) Cit**-**MNPs and (c) chitosan coated DOX / Cit**-**MNPs**.**

**Fig.S3. TGA analysis for chitosan coated DOX-Cit-MNPs.**

***
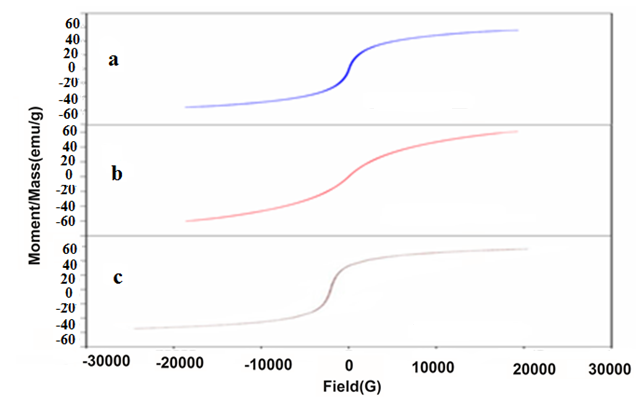

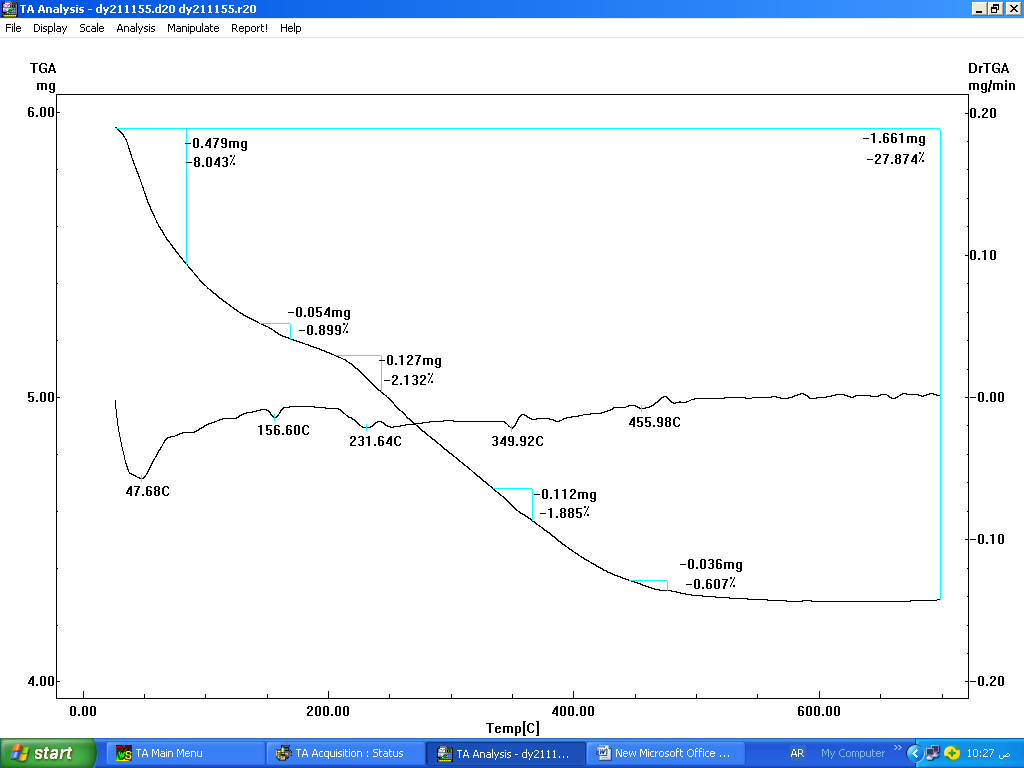
***

**Fig.S4. Magnetization curve of: (a) naked MNPs (b) Cit-MNPs, (c) chitosan coated DOX- Cit-MNPs and.**


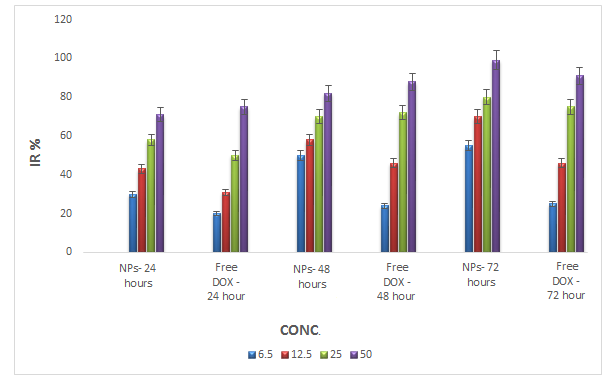


**Fig.S5.** Comparison between inhibition rate (IR %) of Chitosan coated DOX-Cit-MNPs with different concentrations (6.5, 12.5, 25, 50 μg/mL) and free DOX with magnetic directing toward MCF-7 after 24, 48 & 72h by external magnet. The results are expressed as the mean ± standard deviation with (n) = 6 and p <0.001.
